# Translatome data of bundle sheath and mesophyll cells of leaves of the C_4_ species *Flaveria bidentis*

**DOI:** 10.1038/s41597-026-08636-8

**Published:** 2026-11-13

**Authors:** Stefanie Schulze, Peter Westhoff

**Affiliations:** Institut für Entwicklung- und Molekularbiologie der Pflanzen Heinrich-Heine-Universität Universitätsstraße 1, 40225 Düsseldorf, Germany

## Abstract

C_4_ photosynthesis, a specialized form of photosynthesis that uses a CO_2_ concentrating mechanism, evolved more than 60 times independently within various families of the angiosperms. This evolution entailed anatomical and biochemical changes within the plants which were accompanied by a plethora of differentially expressed genes. The differentiation of bundle sheath and mesophyll cells in the leaves was an essential anatomical adaption in many C_4_ species and was accompanied by a differential distribution of photosynthetic processes between these two cell types, whereas in C_3_ species each leaf chlorenchyma cell is able of carrying out all photosynthetic reactions. Leaves of the C_4_ *Asteracean* species *Flaveria bidentis* were used to generate translatome data for bundle sheath and mesophyll cells allowing to elucidate which genes are expressed differentially between these cell types.

## Background & Summary

C_4_ photosynthesis evolved as a specialized form of photosynthesis to cope with various environmental pressures such as high light intensities, heat or drought^1,2^. It originated more than 60 times independently both in monocot and dicot plant genera^3,4^ indicating that the formation of the C_4_ photosynthetic traits must have been genetically relatively easy under favorable conditions. Many of today´s commonly grown crops, e. g. maize, sorghum or sugarcane, conduct C_4_ photosynthesis. Mathematical modeling^5^ taking into account the previously postulated steps from C_3_ to C_4_ photosynthesis^6–9^ could allocate a fitness advantage to each individual step thus explaining the evolutionary trajectory taken.

The division of labour in organizing the photosynthetic processes between bundle sheath and mesophyll cells is the hallmark of C_4_ photosynthesis. Although studies were performed to identify genes that are differentially expressed in the two cell types, most of these studies were performed with monocot C_4_ species of the grass family^10–12^. The leaf anatomy of grasses with their parallel vein architecture allows a relatively easy access to the mechanical separation of bundle sheath strands from mesophyll cells as compared to dicot C_4_ species with their complex vein structure^13,14^.

Mustroph, Zanetti and colleagues designed an experimental strategy for obtaining information on cell-specific translatomes^15–18^. These authors used cell-specific promoters to tag ribosomal complexes in *Arabidopsis thaliana* by expressing a tagged version of the ribosomal protein L18B (RPL18B). This allowed ribosomal complexes to be isolated by using the tag for affinity chromatography and the sequencing of the RNAs bound to those complexes.

In this study we adapted the method for the C_4_ *Astaracean* species *Flaveria bidentis* to obtain bundle sheath and mesophyll specific translatomes. The genus *Flaveria* is one of the few genera that besides C_3_ and C_4_ species contains a large number of species with C_3_-C_4_ intermediate photosynthetic characteristics^19–24^. The genus is therefore often used to study the evolutionary trajectory from C_3_ to C_4_ photosynthesis (e. g.^5,19,25–30^). Having access to a dataset of translatomes of bundle sheath and mesophyll cells of a C_4_ *Flaveria* species allows a more comprehensive view into the extent of differential gene expression in dicot C_4_ photosynthesis and to identify novel C_4_ genes, for instance, for transcription factors or transporters that are hitherto not known.

## Methods

### Generation of transgenic *Flaveria bidentis* plants with bundle sheath- or mesophyll- specifically tagged ribosomes

The method for the generation and isolation of cell-specific ribosomes using a tagged ribosomal protein L18 (RPL18) was described in great detail for *Arabidopsis thaliana*^15–18^ and was adapted for *Flaveria bidentis* as follows.

The chimeric tagging constructs were driven either by the *GLDP*A promoter of the gene encoding the PA subunit of glycine decarboxylase of *Flaveria trinervia* for bundle sheath specific expression^31,32^ or the *ppc*A promoter of the gene for the C_4_ phospho*enol*pyruvate carboxylase A of *F. trinervia* for mesophyll specific expression^25,33–35^. Although these promoters drive expression predominantly in bundle sheath and mesophyll cells respectively additional expression was shown previously in the guard cells in case of the* GLDPA* promoter^31,32^. Figure 1 shows a visual representation of the expression pattern of a nuclear localized H2B-YFP (yellow fluorescent protein) driven by these promoters in transgenic *F. bidentis* plants. The transgenic plants for leaf cross-sections were grown from seeds with either *Ft*GLDPA-H2B-YFP or *Ft*ppcA-H2B-YFP or in the pBI121 vector as described previously^31,32^.

**Fig. 1:**
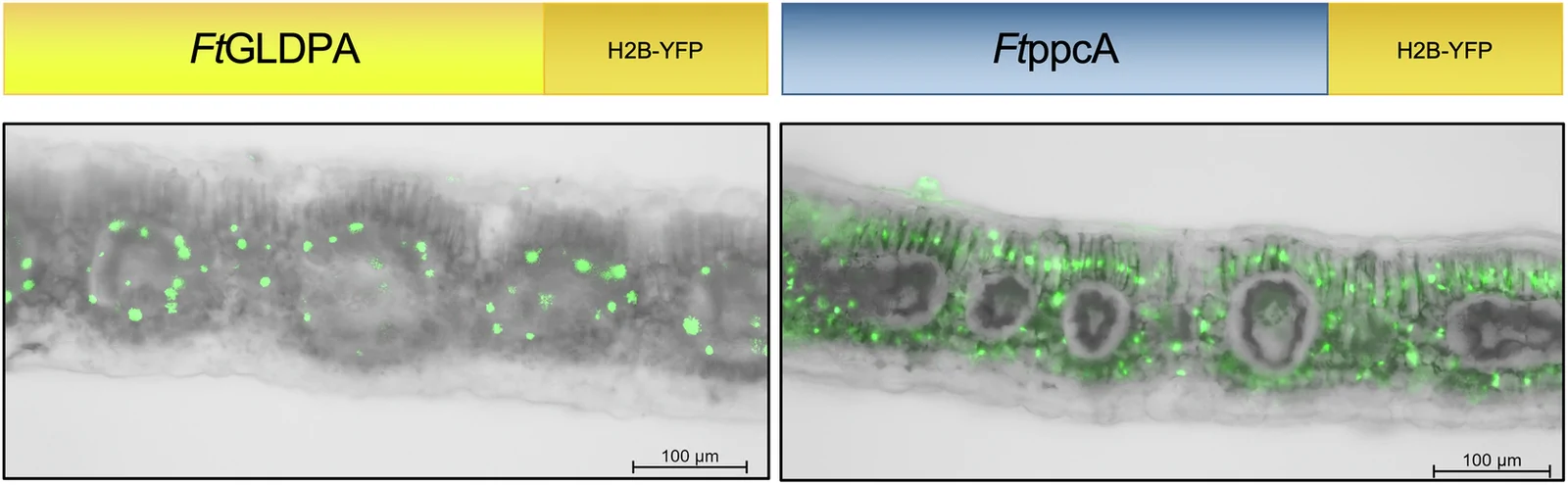
Visual representation of the expression pattern of the promoters used for bundle sheath and mesophyll expression. Illustration of the expression pattern of a nuclear localized yellow fluorescent protein (H2B-YFP) driven by the promoters of the glycine decarboxylase PA subunit (GLDPA, left) or the phospho*enol*pyruvate carboxylase A (ppcA, right) of *Flaveria trinervia* in handmade cross-sections of the 4^th^ leaves of adult transgenic *Flaveria bidentis* plants. Images were taken with an Axio Imager M2 (Carl Zeiss, Jena, Germany) taking an automatically merged image with the AxioCamICc5 camera for brightfield and fluorescence (reflector HE Yellow Fluorescent Protein, excitation 488–512 nm, emission 520–550 nm) respectively using the ZEN 2012 software (Carl Zeiss, Jena, Germany). Scale bars represent 100 µm and were added using the version ZEN 3.13 (Carl Zeiss, Jena, Germany) in the ZEN Lite version.

In order to isolate cell specific ribosome-bound mRNAs two constructs were generated with either the *Ft*GLDPA (Fig. 2) or *Ft*ppcA (Fig. 3) promoters and transformed into *F. bidentis* plants. The coding sequence of a RPL18 homologue of *F. bidentis* was identified by a Blast search using the RPL18B sequence of *Arabidopsis thaliana* (At3g0559) against the transcriptome data of *F. bidentis*^30^. The full sequence of *Fb*RPL18 has been submitted to the NCBI GenBank under PZ378741. In addition, a tag consisting of six histidines (His(6)) and a FLAG sequence, separated by spacers, was fused in frame to the N-terminus of *Fb*RPL18^15–18^ using overlapping primers (Table 1). The detailed sequences of these chimeric constructs are depicted in Figs. 2 and 3. The correctness of the chimeric His(6)-FLAG-tagged *Fb*RPL18 was verified by sequencing (LGC Genomics, Berlin, Germany) before insertion into the binary vector pBI121^36,37^ containing either the *Ft*GLDPA^31^ or *Ft*ppcA^35^ promoter using restriction enzymes. *Agrobacterium tumefaciens* mediated transformation of *F. bidentis* was performed according to the protocol developed by Chitty *et al*^38^.

**Fig. 2:**
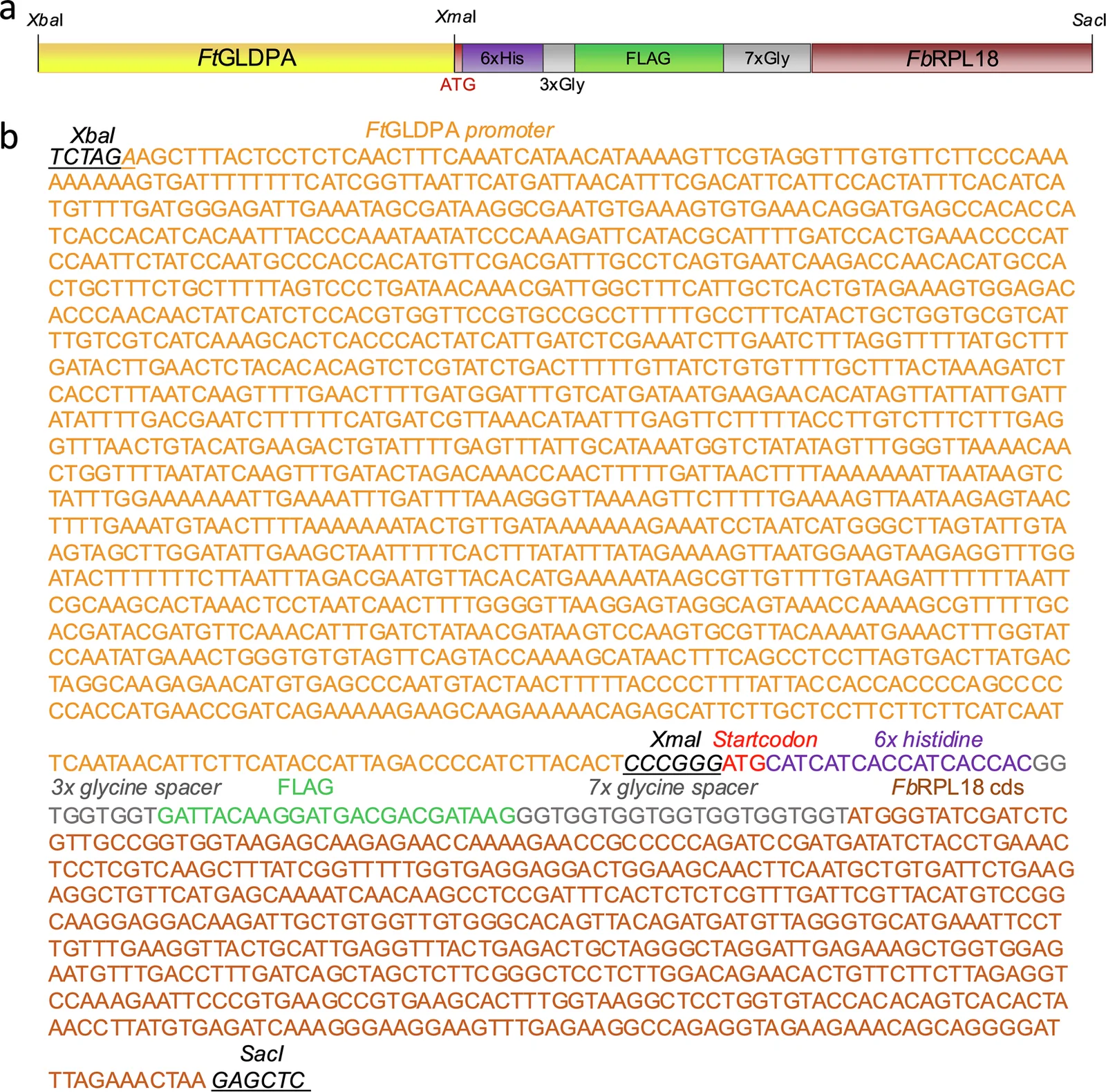
Chimeric construct for bundle sheath specifically tagged ribosomes. (**a**) Schematic representation of the sequence of the His(6)-FLAG tagged *FbRPL18* coding sequence fused with the GLDPA promoter of *Flaveria trinervia* (not to scale). (**b**) Detailed sequence of the *Ft*GLDPA promoter fused with the tagged *Fb*RPL18 cds. Restriction sites are in italics and underlined, the promoter sequence is orange, the startcodon 5ʹ of the tag red, the His(6) purple, the FLAG tag green, the glycine spacers gray and the cds for *Fb*RPL18 brown.

**Fig. 3:**
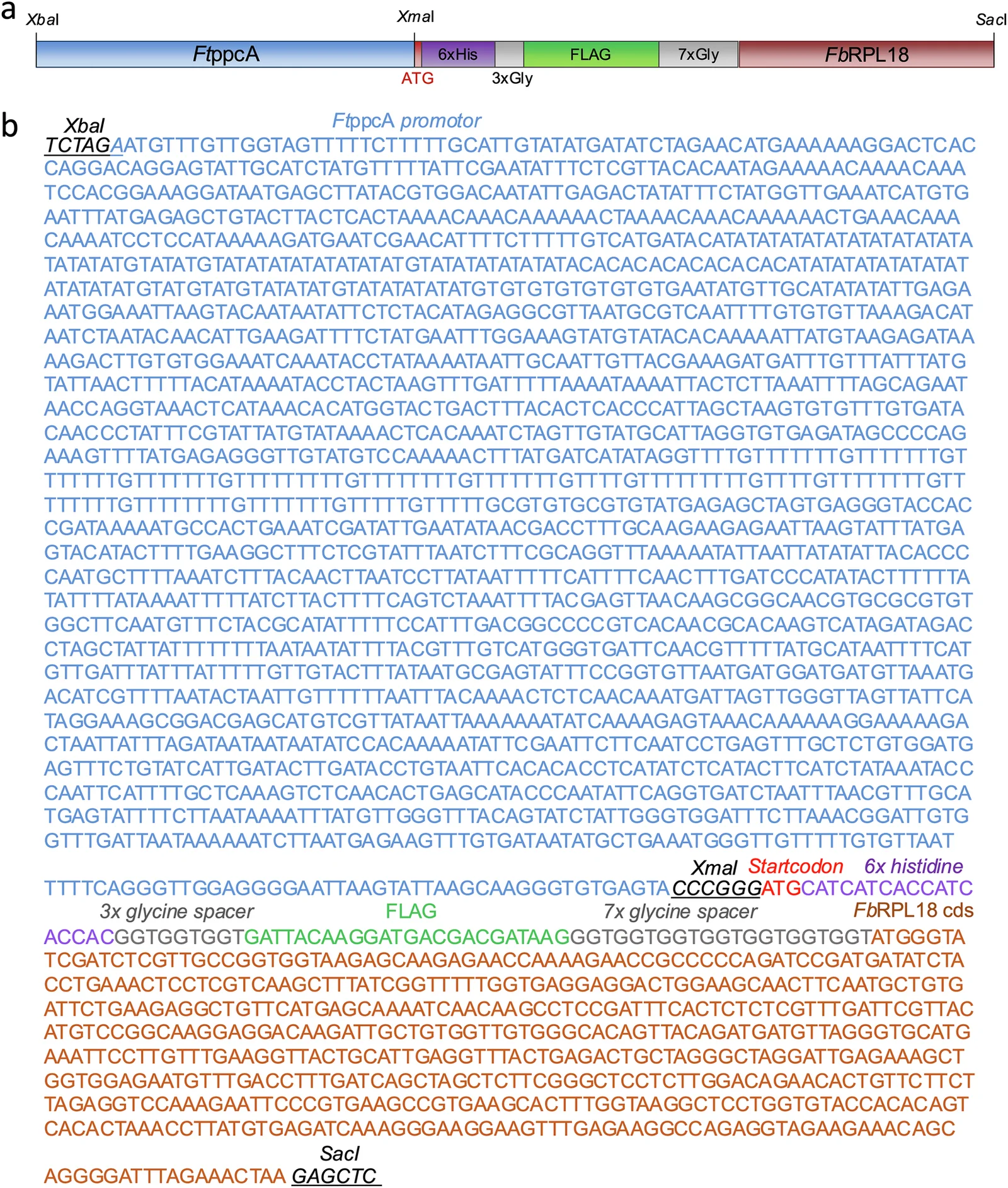
Chimeric construct for mesophyll specifically tagged ribosomes. (**a**) Schematic representation of the sequence of the His(6)-FLAG tagged *FbRPL18* coding sequence fused with the ppcA promoter of *Flaveria trinervia* (not to scale). (**b**) Detailed sequence of the *Ft*ppcA promoter fused with the tagged *Fb*RPL18 cds. Restriction sites are in italics and underlined, the promoter sequence is blue, the startcodon 5´ of the tag red, the His(6) purple, the FLAG tag green, the glycine spacers gray and the cds for *Fb*RPL18 brown.

**Table 1:** Primers used in this study and their purpose.

primer name	Sequence 5′ to 3′	purpose
FbRPL18_fw	ATGGGTATCGATCTCGTTGC	Generation of tagged FbRPL18
FbRPL18_rv	TTAGTTTCTAAATCCCCTGC	Generation of tagged FbRPL18
FbRPL18_rv_*Sac*I	TAT*GAGCTC*TTAGTTTCTAAATCCCCTGC	Generation of tagged FbRPL18
Tag1_FbRPL18_fw	ATGACGACGATAAGGGTGGTGGTGGTGGTGGTGGTATGGGTATCGATCTCGTTGC	Generation of tagged FbRPL18
Tag2_FbRPL18_fw_*Xma*I	TAT*CCCGGG*ATGCATCATCACCATCACCACGGTGGTGGTGATTACAAGGATGACGACGATAAGGGTGGT	Generation of tagged FbRPL18
Actin_fw	AATGGAAGCTGCTGGTATTCA	Internal control in qRT-PCR
Actin_rv	CAACCACCTTGATCTTCATGC	Internal control in qRT-PCR
PPDK_fw	CAAGTTCGTGCGATCTTTCA	Mesophyll marker gene in qRT-PCR
PPDK_rv	AGCAGCTCGAGGAATCTCAA	Mesophyll marker gene in qRT-PCR
GLDPA_fw	TGGCTGATAAGTGGACCAAA	Bundlesheath marker gene in qRT-PCR
GLDPA_rv	CCAGAATTTAGCAGCACGAA	Bundlesheath marker gene in qRT-PCR

Transgenic T0 plants were identified by their Kanamycin resistance, and the expression of the tagged *Fb*RPL18 protein was tested by immunoblottting. Total leaf protein was isolated according to^39^ using approximately 500 mg ground freshly frozen leaf material and 1 ml buffer. As no direct comparison between samples was necessary equal volumes (10 µl each) rather than equal protein quantities were loaded together with a PageRuler Prestained Protein Ladder (Thermo Fisher Scientific, Waltham, USA) on a 10% polyacrylamide SDS gel^40^ and separated by electrophoresis, followed by a transfer to a nitrocellulose membrane (Protran BA85, 0.45 µm, Schleicher & Schuell, Dassel, Germany). A primary anti-DYKDDDK-antibody (Clontech Laboratories, Mountain View, USA) in combination with a Horseradish peroxidase (HRP)-conjugated secondary antibody (Sigma-Aldrich, St. Louis, USA) was used to visualize the tagged protein. The signal was recorded with a ImageQuant^TM^ LAS-4000 mini CDC camera system (GE Healthcare, Piscawa, USA) using an enhanced chemiluminescent HRP-substrate (Thermo Fisher Scientific, Waltham, USA).

Plants expressing the tagged proteins were used for seed production and three T1 plants per construct were used for the subsequent RNA isolation after confirming that they still express the tagged protein.

### Polysome purification and RNA isolation

The fourth and fifth leaves of three individual transgenic *F. bidentis* T1 plants (labelled BS1, BS5 and BS13 for bundle sheath and M5, M8 and M14 for mesophyll, Fig. 4a) for each cell type were harvested at the same time point. All plants were grown next to each other in the greenhouse of the Heinrich-Heine-University at Duesseldorf (Germany) with additional light for 16 h per day under the same conditions. Harvesting was done in the middle of the light period before the onset of flowering.

**Fig. 4:**
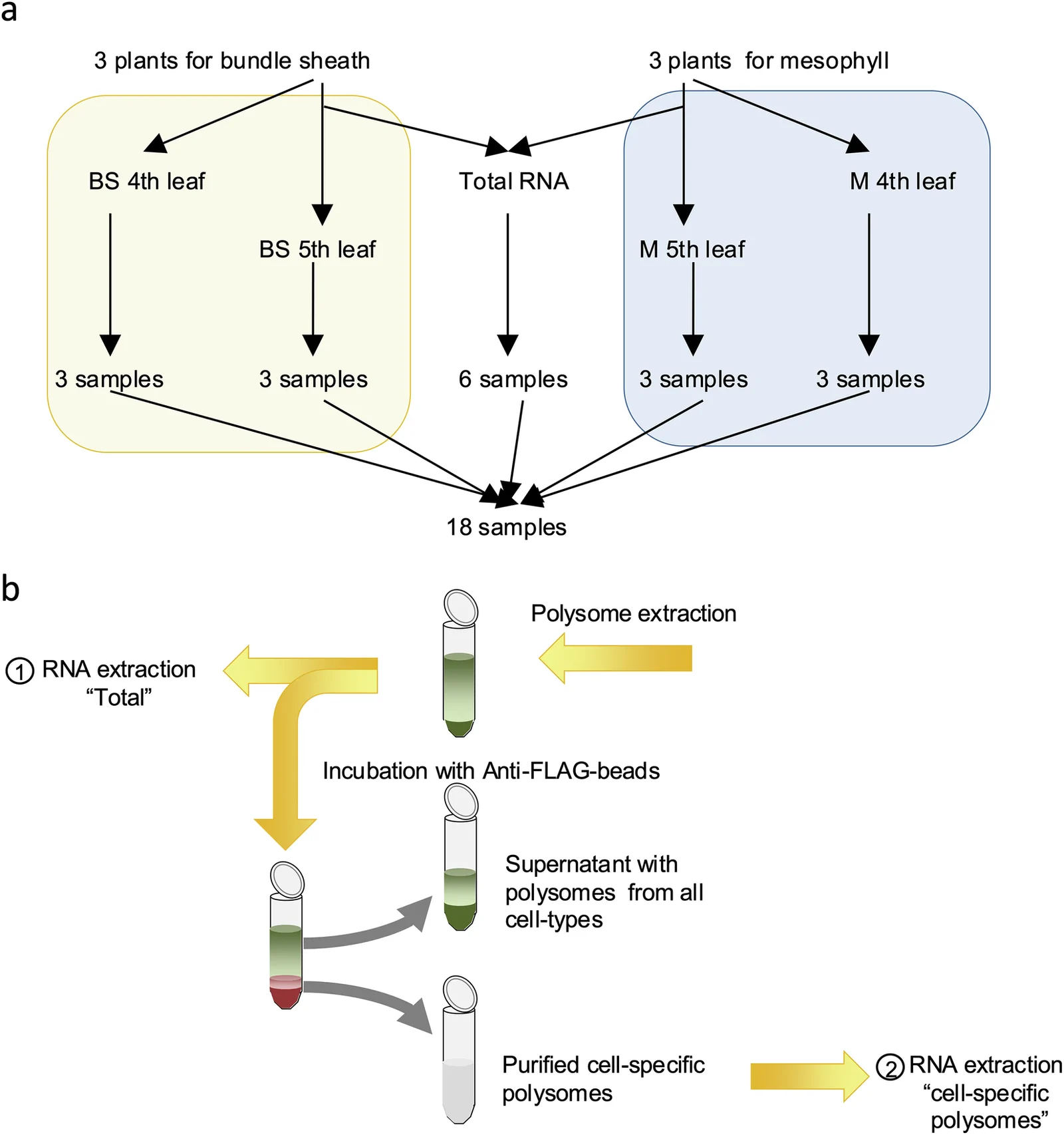
Overview of sample collection during leaf harvest and polysome purification for RNA isolation. (**a**) Schematic representation of the samples collected and used für RNA isolation and sequencing. (**b**) Overview of the procedure to isolate cell type-specific polysomes and the steps at which samples for RNA isolation for the preparation of total RNA (1) and cell type-specific RNA (2) were taken.

Cell-specific polysomes were isolated as described in^18^ up to step 10 of subsection 3.3. RNA isolation followed reference^15^ adding two volumes 8 M guanidine HCl and three volumes 100% ethanol to either the eluate and to an aliquot that was kept from the supernatant before adding the EZview^TM^ ANTI-FLAG^®^-agarose beads (Sigma, St. Louis, USA) to isolate RNA (Figs. 4a and 4b). After washing and resuspension in H_2_O the RNA was purified by using the RNeasy Plant Mini Kit (Qiagen, Hilden, Germany) including an on-column DNAse digestion as stated in the manual of the RNeasy Plant Mini Kit.

RNA quantity was measured with the NanoDrop ND-1000 (Thermo Fisher Scientific, Waltham, USA) and quality was checked with a Pico Chip on an Agilent 2100 bioanalyzer (Agilent, Santa Clara; USA, Fig. 5).

**Fig. 5:**
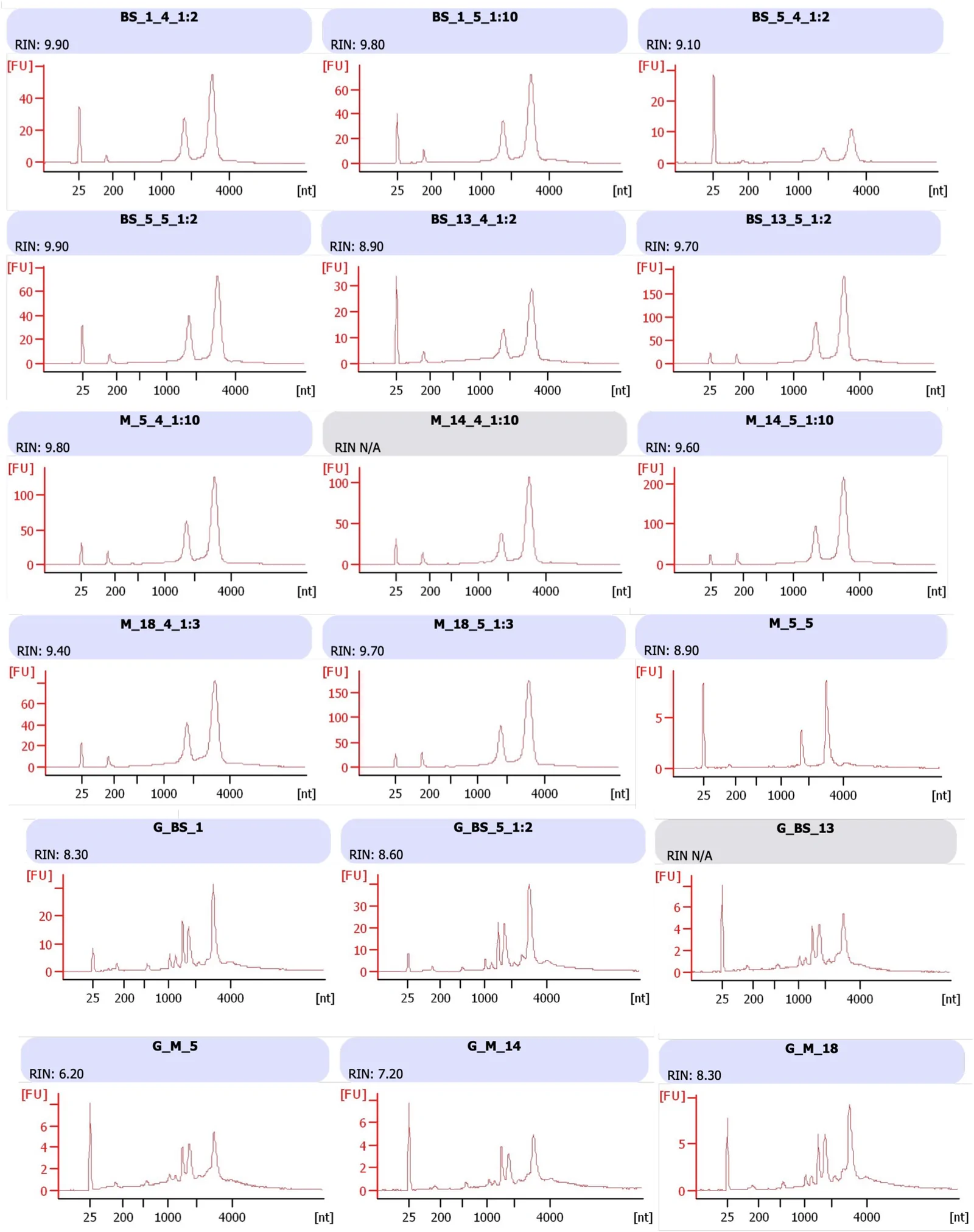
Quality control of RNA samples for sequencing. Quality control of the RNA isolated from purified polysomes with an Agilent Pico chip on an Agilent 2100 bioanalyzer.

### Quantitative Reverse Transcription PCR (qRT-PCR)

For each 5^th^ leaf sample (BS1, BS5, BS13, M5, M8 and M14) 20 ng of RNA was reverse transcribed with the QuantiTect Reverse Transcription Kit (Qiagen, Hilden, Germany) according to the manufacturer´s instructions. A 100-fold dilution of the cDNA was used for quantitative Reverse Transcription PCR (qRT-PCR) in combination with the KAPA SYBR® FAST qPCR Kit (KAPA Biosystems, Wilmington, USA) in a 7500 Fast Real Time PCR machine (Applied Biosystems, now Thermo Fisher Scientic, Waltham, USA). The delta-delta-C_t_ (2^−DDCt^) method^41^ was used for quantification. A sample of total leaf RNA from plant M5 (Total M5) was randomly selected from the total RNA samples and set as reference and actin was used for internal normalization. Primers can be found in Table 1.

### Library preparation and Illumina sequencing

Both library preparation and Illumina sequencing were performed as a paid service at the Genomics & Transcriptomics Laboratory (GTL) of the Biologisch-Medizinisches Forschungszentrum (BMFZ) at the Heinrich-Heine-University at Duesseldorf. Libraries were prepared with the NuGen 0351-32 Ovation® Arabidopsis RNA-Seq System 1–16 (NuGen, San Carlos, USA) with 10 ng of purified cell-specific translatome RNA each for the bundle sheath and mesophyll samples and with 10 ng purified total RNA for the total leaf sample. Quality and quantity of the libraries were analyzed with a Qubit DNA HS Assay and a Fragment Analyzer High Sensitivity NGS Fragment Kit (1 bp – 6000 bp), DNF-474 (Agilent, Santa Clara, USA). Paired end reads (150 + 150) were generated with an Illumina HiSeq. 3000 machine (Illumina Inc. San Diego, USA), using the PE-410-1001 HiSeq® 3000/4000 PE Cluster Kit and the FC-410–1003 HiSeq® 3000/4000 SBS Kit (300 cycles) (Illumina Inc., San Diego, USA).

### Identification of differential expressed genes with CLC Genomics Server

The generated reads were imported into CLC Genomics Server 21.0.1 (Qiagen, Hilden, Germany) and trimmed for quality control using standard settings. The RNA-Seq operation of the program was used for each of the samples mapping the reads to the transcripts which were annotated for *F. bidentis* and provided in the supplemental data sets of^42^ as a reference. The following parameters were used: Reference type = One reference sequence per transcript; Enable handling of spike-in controls = Do not use spike-in controls; Mismatch cost = 2; Insertion cost = 3; Deletion cost = 3; Length fraction = 0.8; Similarity fraction = 0.8; Global alignment = No; Auto-detect paired distances = Yes; Strand specific = Both; Library type = Bulk; Maximum number of hits for a read = 10; Count paired reads as two = No; Expression value = TPM; Minimum read count fusion gene table = 5; Create reads track = Yes; Create report = Yes; Create fusion gene table = No; Create list of unmapped reads = No.

A principal component analysis (PCA) using all mesophyll and bundle sheath RNA-Seq samples mapped against the *F. bidentis* transcripts was performed with CLC Genomics Server 21.0.1 (Qiagen, Hilden, Germany) to identify samples that cluster together (Fig. 6).

**Fig. 6:**
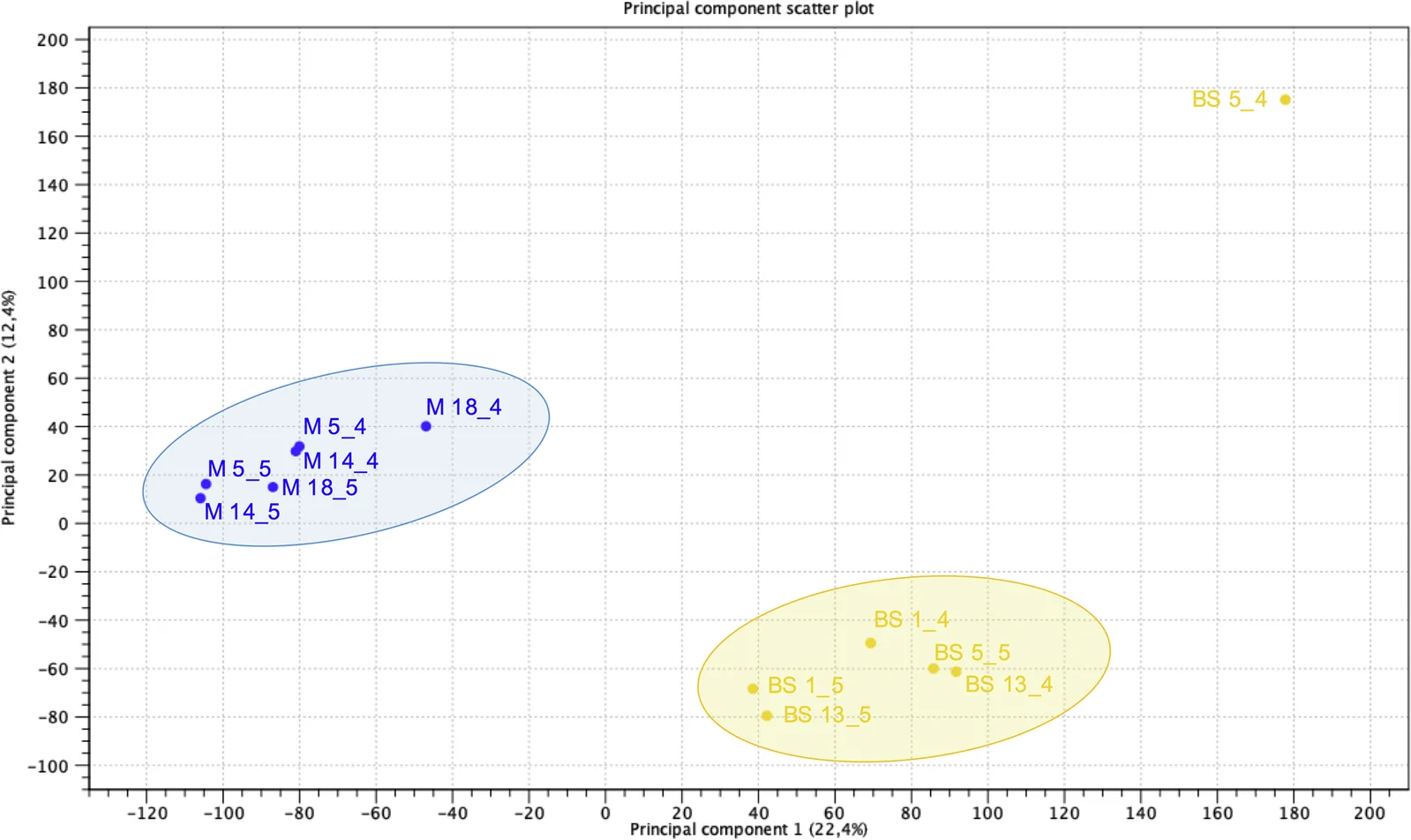
Principal component analysis (PCA) of bundle sheath and mesophyll samples. Principal component analysis of the sequenced RNA-Seq samples for mesophyll (blue) and bundle sheath (yellow). The analysis was conducted with CLC Genomics Server 21.0.1 (Qiagen, Hilden, Germany) with the tool “PCA for RNA-Seq” Principal component 1 equals 22.4 % and principal component equals 12.4 %.

Genes differentially expressed between mesophyll and bundle sheath cells were identified by applying the “Differential Expression in Two Groups” tool of the CLC Genomics Server 21.0.1 (Qiagen, Hilden, Germany). All mesophyll samples were used as group 1 and all bundle sheath samples minus BS 5_4 were used as group 2. BS 5_4 was excluded due to the PCA showing that this sample did not cluster with the other bundle sheath samples (see Fig. 6). As settings “Technology = Whole transcriptome RNA-Seq and Filter on average expression for FDR correction = Yes” were chosen. The complete table generated in this analysis is available as Supplemental File 1^43^.

## Data Records

All generated RNA-Seq datasets are available at the NBCI Sequence Read Archive (SRA) in the SRA study SRP698054^44^ (https://www.ncbi.nlm.nih.gov/sra/SRP698054) with individual fastq files for all 18 datasets (Accessions SRX33238385 - SRX33238402). Metadata for the datasets is also available with each sample in the study.

The cds sequence for *Fb*RPL18 has been submitted to the NCBI GenBank under the Accession Number PZ378741^45^.

Supplemental File 1^43^ is available at https://doi.org/10.6084/m9.figshare.32187051.

## Data Overview

A principal component analysis (PCA, Fig. 6) was conducted with the datasets as described above. Principal component 1 (22.4%) and principal component 2 (12.4%) separate samples from bundle sheath (yellow) and mesophyll (blue) clearly from each other and all samples from each cell type cluster together with the exception of sample BS5_4. The data from the 4^th^ leaf of bundle sheath plant 5, did not cluster with the other bundle sheath samples and were thus excluded from the subsequent initial check for differentially expressed genes.

A dataset of differentially expressed genes (Supplemental File 1^43^) was generated as described above. A volcano plot (Fig. 7) was used to visualize the pattern of differentially expressed genes between bundle sheath and mesophyll cells. Both show that the datasets generated here can be used for investigating the extent of differential gene expression in the bundle sheath and mesophyll cells of *F. bidentis*.

**Fig. 7:**
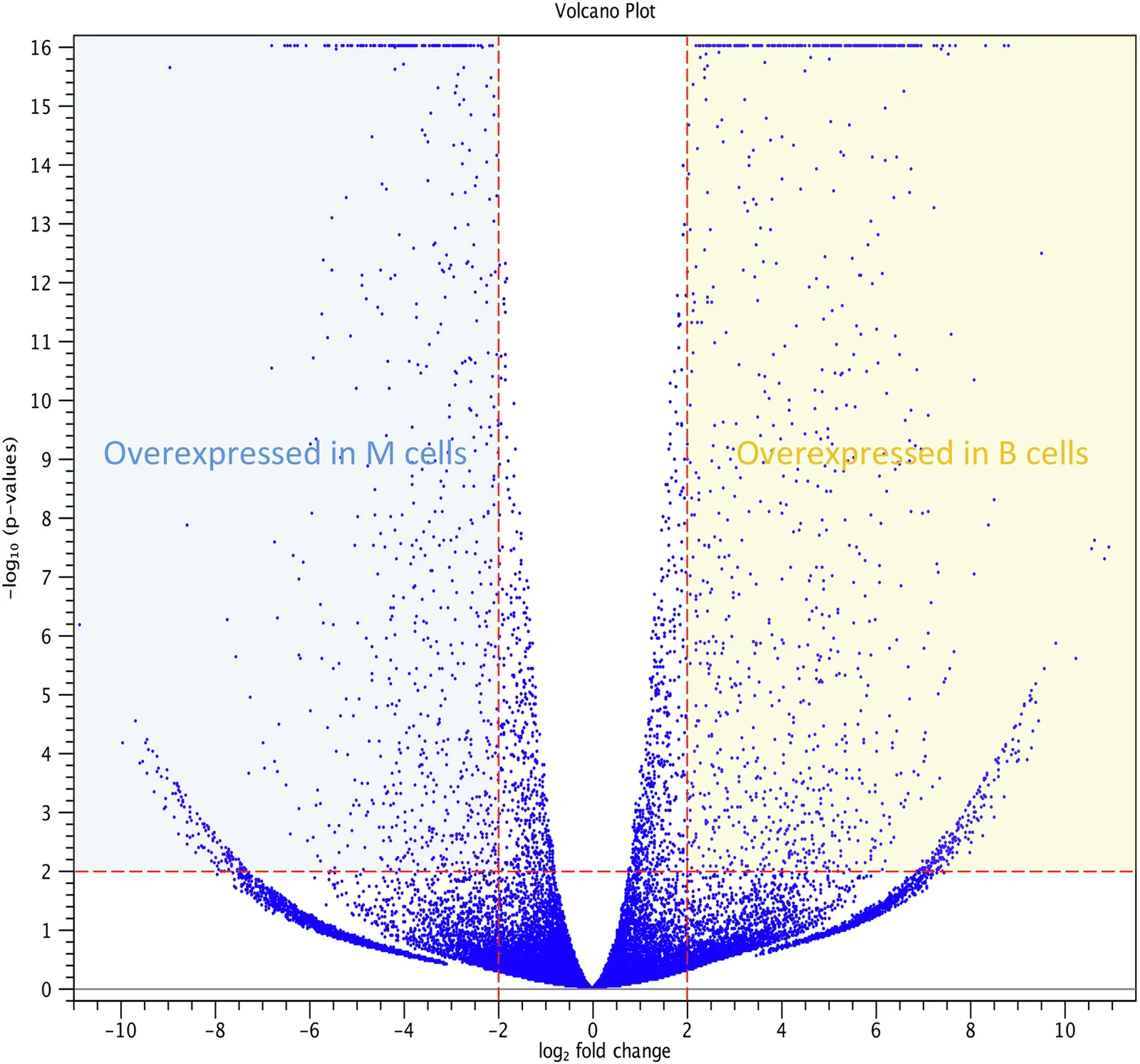
Volcano plot of bundle sheath and mesophyll samples to visualize differential gene expression. Volcano plot of the genes of bundle sheath and mesophyll translatomes. Genes in the blue and yellow area mark those that are higher expressed in mesophyll (log 2 fold change ≥- 2 and log_10_ of the p-value ≥ 2) and bundle sheath cells (log 2 fold change ≥ 2 and log_10_ of the p-value ≥ 2) respectively. The plot was generated with CLC Genomics Server 21.0.1 (Qiagen, Hilden, Germany).

## Technical Validation

### Validation that transgenic plants express the tagged RPL18 protein

After generating the transgenic *F. bidentis* plants total leaf protein was isolated and an antibody against the inserted FLAG-tagged RPL18 protein was used to show the successful expression in each independent plant line used in this study. Figure 8a shows the blot for the T1 plants which were used for polysome purification and subsequent RNA isolation.

**Fig. 8:**
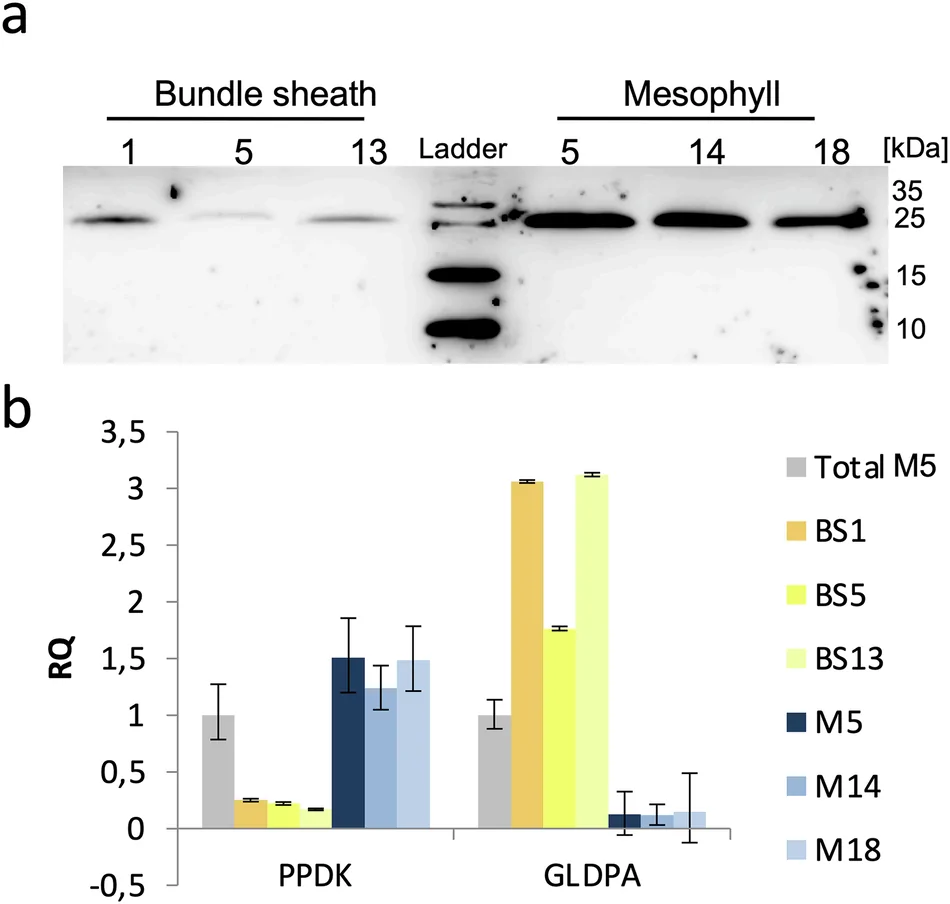
Validation of expression of the tagged proteins *in planta* and cell specificity of the isolated RNAs. (**a**) Immunoblot signal against the FLAG-tag introduced into the plants to confirm expression *in planta*. Numbers represent the respective individual plant. (**b**) qRT-PCR was performed to confirm separation of the RNAs according to cell-type using the samples from the 5^th^ leaves each. Pyruvate orthophosphate dikinase (PPDK) was used as a reference transcript for mesophyll cells and the P subunit of the glycine decarboxylase (GLDPA) as a reference transcript for bundle sheath cells. Total RNA of plant M5 (Total M5) of a was used randomly to normalise against. Numbers represent the respective individual plant line.

### Validation that separation of polysomes worked and the subsequently isolated RNAs are either bundle sheath or mesophyll specific

After the cell-specific polysome purification and subsequent RNA isolation of the samples described in Fig. 4a, qRT-PCR analyses with known differentially expressed genes were performed to test whether the isolation of cell-specific polysomes was successful (Fig. 8b). Transcripts for pyruvate orthophosphate dikinase (PPDK) were used as a reference for a mesophyll-specific transcript and RNAs encoding the PA subunit of glycine decarboxylase (GLDPA) served as reference for bundle sheath specificity^28,30,42^. Actin transcript amounts were used to normalize for equal expression in all samples, and total RNA from plant M5 (mesophyll 5^th^ leaf) was randomly selected as a reference and set to 1. In case of PPDK transcripts, which are expected to accumulate preferentially in mesophyll cells, all three bundle sheath samples showed relative quantities (RQs) between 0.1689 and 0.2498 with a mean of 0.2131, whereas the mesophyll samples showed RQs between 1.2359 and 1.507 with a mean of 1.4092 compared to the total RNA sample (Fig. 8b). For *GLDPA*, whose expression is restricted to the bundle sheath cells^31^, the bundle sheath samples had RQs between 1.7645 and 3.1216 with a mean of 2.649 and the mesophyll samples between 0.119 and 0.1507 with a mean of 0.1306 compared to the total RNA sample (Fig. 8b). These results indicated that the isolation of cell specific polysomes was successful, and all isolated RNA samples were subsequently subjected to RNA-Seq analysis.

## Data Availability

The raw data for this study have been submitted to the NCBI Sequence Read Archive (SRA) in the study SRP698054^44^ (https://www.ncbi.nlm.nih.gov/sra/SRP698054) with the fastq files for each sample (Accessions SRX33238385 - SRX33238402).The cds sequence for FbRPL18 has been submitted to the NCBI GenBank under the Accession Number PZ378741^45^ (https://identifiers.org/nucleotide:PZ378741). Supplemental File 1^43^ is available at https://doi.org/10.6084/m9.figshare.32187051.

## Code availability

No in house code was used.
